# The Effect of Item Strength on Retrieval-Induced Forgetting in Social Interaction

**DOI:** 10.3390/bs14100950

**Published:** 2024-10-15

**Authors:** Yue Chu, Hui Xu, Weihai Tang, Xiping Liu

**Affiliations:** Faculty of Psychology, Tianjin Normal University, Courtyard 393, Binshuixi Road, Xiqing District, Tianjin 300387, China; cypsy11@sina.com (Y.C.); 2000340008@stu.tjnu.edu.cn (H.X.); twhpsy@126.com (W.T.)

**Keywords:** retrieval-induced forgetting, socially shared retrieval-induced forgetting, inhibition, item strength

## Abstract

Retrieval-induced forgetting (RIF) refers to the phenomenon in which people retrieve information, but forget related information. RIF also occurs when people interact with each other. In social interactions, information recalled by the speaker can lead the listener to forget related information, a phenomenon known as socially shared retrieval-induced forgetting (SS-RIF). How does SS-RIF occur? Does it have similar mechanisms to RIF? By observing SS-RIF and RIF with different item strengths, this study investigated the mechanisms of SS-RIF. Item strength was manipulated based on exemplar taxonomic frequency, with high-frequency exemplars designated as strong items and low-frequency exemplars as weak. Experiment 1 found that only strong items exhibited SS-RIF and RIF, while weak items did not exhibit either SS-RIF or RIF. In Experiment 2, participants were asked to restudy the materials, and only the strong items still exhibited SS-RIF and RIF. Additionally, the magnitude of SS-RIF observed in Experiment 2 was similar to that observed in Experiment 1, as well as the performance on RIF. The findings of this study provide evidence for the inhibition mechanism of both SS-RIF and RIF, indicating a shared underlying mechanism.

## 1. Introduction

Retrieval-induced forgetting (RIF) refers to when people retrieve a piece of information in their memory, but forget the information related to it [1]. It has been established as a robust phenomenon through extensive research [2,3,4]. If two or more people share the retrieval process, would RIF still occur? Does the information retrieved by speakers influence the memory of listeners in social interaction? Cuc et al. [5] initially focused on this issue, finding that when speakers retrieved parts of the information, listeners also experienced forgetting of the related information. This type of forgetting by listeners is called socially shared retrieval-induced forgetting (SS-RIF).

The classic RIF paradigm involves four stages: study, retrieval practice, distractor, and test phases. In the study stage, participants learn “category–exemplar” pairs. Retrieval practice targets certain category exemplars, creating three item types: retrieved (Rp+), unretrieved, but category-related (Rp−), and unrelated (Nrp). The test phase assesses recall of all studied exemplars, revealing that Rp− items are recalled less than Nrp items [1]. SS-RIF research, typically conducted in pairs with a speaker and a listener, follows a four-phase paradigm. Unlike RIF, the speaker performs retrieval practice while the listener evaluates the speaker’s accuracy, with both sharing the same Rp+, Rp−, and Nrp items [5].

Anderson et al. [1] initially introduced the inhibition theory for RIF, where non-target items are inhibited during retrieval practice to reduce competition with targets, weakening their memory representations and leading to impaired recall on subsequent tests. The inhibition mechanism aims to resolve competition from non-target items. More recently, the blocking theory has emerged, proposing that retrieval practice strengthens the association of target items and category cues, causing non-target items to be blocked during testing [6,7]. The scholarly community is divided, with some studies supporting inhibition alone and others backing blocking, each questioning the other’s validity [8]. While most studies attribute RIF to a single mechanism, the “two-factor” theory suggests that both inhibition and blocking contribute to RIF [9,10]. The “two-factor” theory, while comprehensive, is a concept that has yet to be precisely articulated. It raises unresolved questions, such as whether both mechanisms operate simultaneously in all RIF instances, their respective contributions, and the conditions under which they act.

Interference dependence is a key aspect of RIF supporting the inhibition theory, where non-target items are inhibited during retrieval practice only when they compete with target items, with greater competition leading to greater RIF [11]. Studies on item strength have been crucial in demonstrating this dependence. Researchers typically manipulate item strength based on the taxonomic frequency of exemplars within their category, with those exemplars with higher taxonomic frequencies as strong items and those with lower taxonomic frequencies as weak items [1,12]. Inhibition theory suggests RIF is limited to strong items that compete more intensely, requiring the activation of inhibitory processes to resolve this competition. Blocking theory, however, predicts RIF in both strong and weak items, as retrieval practice strengthens item–cue associations, potentially blocking non-target recall regardless of initial strength. These contrasting predictions distinguish the mechanisms underlying RIF. Anderson et al. [1] observed that RIF occurred only with strong items, and there was no RIF with weak items. Migueles and García-Bajo [12] replicated these findings. Both of these studies employed cued recall within the RIF paradigm. Beyond cued recall, other researchers have noted similar results using different test formats. Bai and Liu [13] controlled for output interference by using category-plus-stem recall and discovered that RIF also occurred only for strong items. Additionally, Reppa et al. [14] obtained consistent results in the context of recognition tests. Collectively, these findings lend robust support to the inhibition mechanism.

Some studies on item strength diverge from the “interference dependence” prediction. Williams and Zacks [15] replicated Anderson et al.’s work [1], finding equal RIF magnitudes for both strong and weak items, challenging the inhibition mechanism. Jakab and Raaijmakers [16] defined strong items by recall performance and examined RIF effect through item position and numbers of study sessions, finding no item strength influence on RIF. Explaining these findings requires further consideration.

The type of test in the RIF paradigm influences the contribution of inhibition and blocking mechanisms. In recognition tests or category-plus-stem recall, the “strength” effect is minimized, reducing the blocking mechanism’s impact [7,17]. These conditions have consistently supported the inhibition mechanism through interference dependence. The item strength debate mainly arises in cued recall studies, where Rp− items face both inhibition during retrieval and blocking during testing, allowing both mechanisms to operate [8]. So far, we have reviewed studies on the effect of item strength on RIF. Despite varying findings, it is worth noting that interference dependence is a technique used to explore both inhibition and blocking theories. The occurrence of RIF only in strong items aligns with the predictions of the inhibition theory, presenting a challenge for the blocking theory to account for this phenomenon. It is crucial to acknowledge that this does not invalidate the blocking mechanism; rather, it highlights the complexity of RIF mechanisms that remain a subject of ongoing scholarly debate.

Studies have extensively examined RIF mechanisms, yet SS-RIF has received less attention. SS-RIF, a variant of RIF, is often presumed to share RIF’s underlying mechanisms [18,19]. However, this assumption requires scrutiny due to the distinct roles of speakers and listeners, evident in both laboratory and daily interactions. In labs, speakers retrieve information based on cues, while listeners evaluate responses, akin to a recognition task [20]. In natural social interactions, listeners may either remain silent while the speaker is speaking (e.g., during a lecture or in a classroom) or actively engage in dialogue with the speaker (e.g., when discussing various topics). These scenarios highlight the disparities in the cognitive processes of listeners and speakers. Consequently, the proposition that listeners and speakers share identical cognitive mechanisms requires additional empirical support. Some researchers have explored the mechanisms of SS-RIF and discovered that RIF mechanisms differ between listeners and speakers.

Abel and Bäuml [20] first explored SS-RIF mechanisms using the two-factor theory, finding equivalent RIF and SS-RIF magnitudes in category-cued recall, but smaller SS-RIF in recognition tests. They suggested that speakers experienced greater inhibition and less blocking, whereas listeners experienced greater blocking and less inhibition. However, this conclusion needs scrutiny: not all RIF instances may involve both mechanisms. If both were always at play, weak items should consistently show RIF across cued recall. Zhang et al. [21] found RIF in speakers with high inhibition and SS-RIF in listeners with low inhibition, indicating potential RIF and SS-RIF mechanism divergence. Thus, given task differences in the retrieval practice phase and mixed findings on SS-RIF and RIF mechanisms, further research is needed to fully understand SS-RIF processes.

The goal of this study was to examine the mechanisms of SS-RIF, which we analyzed by examining SS-RIF and RIF with different item strengths. To achieve this, we categorized exemplars as strong or weak based on their taxonomic frequencies and employed a cued recall methodology. This approach enabled a more comprehensive examination of the SS-RIF mechanisms. Additionally, we re-evaluated previous controversial findings concerning the RIF of strong and weak items within the context of our study. In Experiment 1, we classified exemplars into strong and weak items based on their taxonomic frequencies and observed their SS-RIF and RIF. If only strong items exhibited SS-RIF and RIF, it would support the inhibition theory. If both strong and weak items exhibited SS-RIF and RIF, it would support the blocking theory or the two-factor theory.

Experiment 2 controlled for item strength by varying the number of study sessions. Restudying items is recognized as an effective method for enhancing item strength [16,22]. Jakab and Raaijmakers [16] conducted a comparative analysis of RIF in conditions where non-target items were studied either once or twice. They hypothesized that the strength of these items would be enhanced following two study sessions, and in line with interference dependence, they anticipated a greater degree of RIF effect. Contrary to the predictions, their findings indicated no significant difference in RIF magnitude between single and dual study conditions. This discrepancy led to a challenge to interference dependence and served as a critical piece of evidence against the inhibition mechanism. To date, their research remains unique in its approach to refuting interference dependence by increasing study sessions to enhance item strength. However, a significant body of previous research has provided ample support for interference dependence [23]. The examination of interference dependence based on the number of study sessions is currently limited to a single study, which may not represent generalizable results. Therefore, in Experiment 2, controlling study sessions aimed to enhance items and assess their effect on RIF and SS-RIF. This strategy both expanded understanding of study sessions effect on RIF, allowing comparison with Jakab and Raaijmakers [16], and examined changes in RIF and SS-RIF for strong and weak items after restudy.

## 2. Experiment 1

### 2.1. Method

#### 2.1.1. Participants

Participants were recruited through advertisements with a financial reward upon completion. The recruitment criteria were non-psychology majors and native Chinese speakers. Demographic information collected included age and gender. A total of 56 university students (10 males and 46 females) were enrolled and organized by sex, with unfamiliar pairs to reduce social influence, resulting in 28 participant groups (5 male and 23 female groups). Random assignment designated one as the speaker and the other as the listener, with mean ages of 20.29 years (SD = 2.21) and 20.25 years (SD = 1.56), respectively. Power analysis using G*Power 3.1 indicated a minimum sample size of 24 for 80% power to detect a medium effect size (f = 0.25) for SS-RIF at α = 0.05. According to this criterion, the experiment required a minimum of 24 listeners, which meant 24 participant groups. This experiment enrolled 28 participant groups, satisfying the requirement.

#### 2.1.2. Materials

##### Category and Exemplar Selection

Referring to Battig and Montague [24], this study utilized eight categories—fruit, sport, crime, disease, occupation, flower, topography, and body—each with 36 two-character Chinese exemplars, ensuring unique first characters for each. Forty-eight psychology students evaluated the taxonomic frequency of each exemplar on a 7-point scale, with “1” as the least frequent and “7” as the most, using the prompt: “When presented with a category, how quickly can you come up with the exemplar?” (e.g., “piano” for “musical instrument”). Exemplars were ranked by average rating. The categories were randomly assigned to two groups: Group A (high-rated, strong items) and Group B (low-rated, weak items), with the top and bottom six exemplars from each category selected, respectively.

##### Study Lists

Eight distinct categories, each with six exemplars, were systematically organized into six study blocks, with each block containing one exemplar per category (e.g., “occupation–doctor”). The experimental design also incorporated two unrelated category–exemplar pairs as fillers, placed before and after the study phase.

##### Retrieval Lists

Participants were tasked with retrieving half of the exemplars from each of four categories, with two selected from Groups A and B. This resulted in 12 exemplars, each retrieved three times. The exemplars were organized into a block, ensuring that no two from the same category were adjacent. Three blocks were created at random for the retrieval list. To ensure balanced retrieval, Groups A and B were randomly divided into sub-groups A1/A2 and B1/B2, forming four unique combinations (retrieval material types), which allowed for equal representation of each exemplar across retrieval conditions.

##### Test Lists

The eight experimental categories were randomly presented in the test phase.

#### 2.1.3. Design

This study aimed to investigate SS-RIF mechanisms and analyze controversial findings on item strength’s impact on RIF. Using a two-factor within-subjects design, we examined recall performance for listeners and speakers, with item strength (strong, weak) and type (Rp+, Rp−, Nrp) as variables and recall accuracy as the dependent variable. Separate statistical analyses for listeners and speakers are detailed in the Results section.

#### 2.1.4. Procedures

##### Study Phase

Two participants sat side by side facing a computer screen, where they were directed to study the presented word pairs with attention, anticipating a subsequent memory test. The study material, consisting of 52 “category–exemplar” pairs, was displayed sequentially with a 500 ms fixation point preceding each 5 s word pair presentation. Randomization occurred within blocks, and filler materials were included at the start and end of the study phase.

##### Retrieval Practice Phase

In this phase, participants were randomly assigned as speakers or listeners. Speakers retrieved exemplars in “occupation–do ( )” format, filling the blanks from studied material and reading aloud. Listeners rated these responses on a 7-point scale (1 = very incorrect, 7 = very correct). After the speaker read the word pair, the listener provided a rating of the speaker’s response. Once the rating was completed, the speaker pressed a button to proceed to the next item. If unable to retrieve, speakers read the screen aloud and tried to respond. If no response was given within 10 s, the system moved to the next item. During retrieval, speakers faced the listener, with only the speaker viewing the screen. After a practice to familiarize themselves with the process, participants began the retrieval phase, aware that their responses were recorded.

##### Distractor Phase

Participants, seated side by side, completed an arithmetic task, recording their responses on answer sheets for a total duration of 3 min, with tasks displayed on a screen.

##### Test Phase

**Figure 1:**
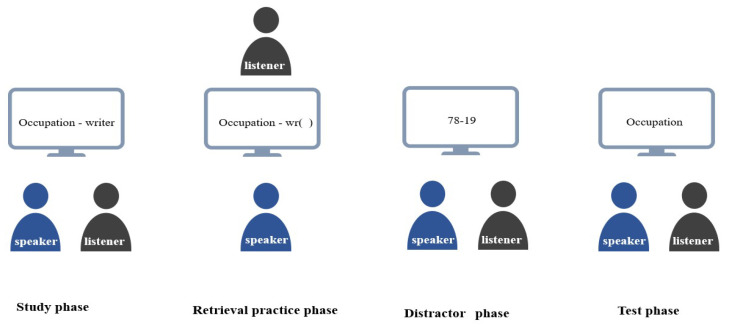
Illustration of the experimental procedures.

Following a distractor task, participants individually recalled exemplars from category cues displayed on a computer screen, each given 90 s per category. The experiment, conducted using E-Prime 2.0 (computer monitor size: 13.5 inches; resolution: 1280 × 768 pixels) lasted about 20 min (see Figure 1).

### 2.2. Results

#### 2.2.1. Recall Performance of Speakers

**Figure 2:**
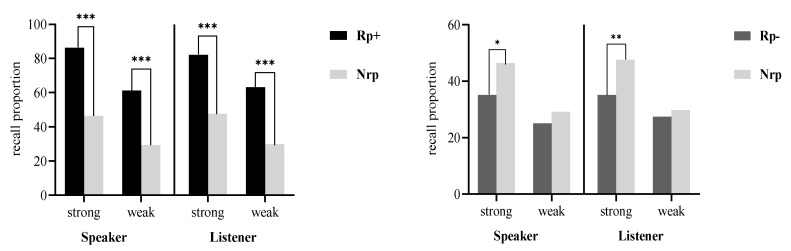
The left subfigure shows the practice effects (Rp+ vs. Nrp); the right subfigure shows the RIF (Rp− vs. Nrp). **Note:** *** *p* < 0.001; ** *p* < 0.01; * *p* < 0.05.

Table 1 shows the recall performance of speakers and listeners in Experiment 1. Figure 2 illustrates the results of the *t*-tests in Experiment 1.

In line with the objectives of this study and referencing the analytical methods of prior research [1,16], we conducted ANOVA and planned comparisons to enhance our analysis and discussion of the findings.

For practice effects, in a 2 (item strength: strong, weak) × 2 (item type: Rp+, Nrp) repeated-measures ANOVA, significant main effects were found for item type (*F* (1, 27) = 109.78, *p* < 0.001, *η_p_^2^* = 0.80) and item strength (*F* (1, 27) = 49.87, *p* < 0.001, *η_p_^2^* = 0.65), with no interaction (*F* (1,27) = 1.82, *p* = 0.188, *η_p_^2^* = 0.06). Planned paired *t*-tests revealed higher recall for Rp+ over Nrp items at both strengths, indicating practice effects (strong: *t* (27) = 9.02, *p* < 0.001, Cohen’s *d* = 1.71; weak: *t* (27) = 7.10, *p* < 0.001, Cohen’s *d* = 1.34).

For RIF, another 2 (item strength: strong, weak) × 2 (item type: Rp−, Nrp) repeated-measures ANOVA showed significant main effects for item type (*F* (1, 27) = 7.82, *p* = 0.0094, *η_p_^2^* = 0.22) and item strength (*F* (1,27) = 28.37, *p* < 0.001, *η_p_^2^* = 0.51), with no interaction (*F* (1, 27) = 1.47, *p* = 0.236, *η_p_^2^* = 0.05). Planned paired *t*-tests indicated RIF only with strong items (*t* (27) = 2.34, *p* = 0.027, Cohen’s *d* = 0.44, B_10_ = 3.97), and no RIF with weak items (*t* (27) = 1.37, *p* = 0.183, Cohen’s *d* = 0.26, B_10_ = 0.83), suggesting RIF is confined to strong items.

#### 2.2.2. Recall Performance of Listeners

For practice effects, a 2 (item strength: strong, weak) × 2 (item type: Rp+, Nrp) repeated-measures ANOVA revealed significant main effects for item type (*F* (1, 27) = 87.72, *p* < 0.001, *η_p_^2^* = 0.76) and item strength (*F* (1, 27) = 27.93, *p* < 0.001, *η_p_^2^* = 0.51), with no interaction (*F* (1, 27) = 0.053, *p* = 0.819, *η_p_^2^* = 0.002.). Planned paired *t*-tests confirmed higher recall for Rp+ over Nrp items at both strengths, indicating practice effects (strong: *t* (27) = 8.50, *p* < 0.001, Cohen’s *d* = 1.61; weak: *t* (27) = 6.96, *p* < 0.001, Cohen’s *d* = 1.31).

For SS-RIF, a 2 (item strength: strong, weak) × 2 (item type: Rp−, Nrp) repeated-measures ANOVA showed a marginally significant main effect for item type (*F* (1, 27) = 4.52, *p* = 0.059, *η_p_^2^* = 0.13) and a significant effect for item strength (*F* (1, 27) = 19.83, *p* < 0.001, *η_p_^2^* = 0.38), with a significant interaction (*F* (1, 27) = 4.83, *p* = 0.037, *η_p_^2^* = 0.15). Planned paired *t*-tests indicated SS-RIF in strong items only (*t* (27) = 2.77, *p* = 0.0099, Cohen’s *d* = 0.52, B_10_ = 9.22), with no effect for weak items (*t* (27) = 0.55, *p* = 0.587, Cohen’s *d* = 0.10, B_10_ = 0.32). This suggests that listeners show SS-RIF with strong items, but that it is absent in weak ones.

### 2.3. Discussion

In Experiment 1, speakers showed RIF with strong items and listeners showed SS-RIF, with these effects absent in weak items. This RIF pattern aligns with previous findings [1,13,25], and the study introduces the novel finding of SS-RIF’s similarity to RIF. The inhibition mechanism implies that non-target items are inhibited during competition with targets, which is less likely with weak items, hence the lack of SS-RIF and RIF. The consistent performance between speakers and listeners supports the inhibition theory as the underlying mechanism for both SS-RIF and RIF.

## 3. Experiment 2

In Experiment 2, based on Experiment 1, the number of study sessions was increased to two. Following the additional study session, memory performance improved for both strong and weak items. That allowed us to observe RIF and SS-RIF for both strong and weak items after restudy. Furthermore, it enabled a comparative analysis of RIF and SS-RIF between Experiment 2 and Experiment 1, thereby providing insights into the influence of restudy on these effects.

### 3.1. Method

#### 3.1.1. Participants

In Experiment 2, the participant recruitment and grouping procedures were consistent with those of Experiment 1. A total of 56 university students, comprising 12 males and 44 females, were recruited to participate. These participants were organized into 6 male groups and 22 female groups. The mean age of the speakers was 20.07 years (SD = 1.46), and the mean age of the listeners was 19.75 years (SD = 1.24).

#### 3.1.2. Materials

The stimulus materials were identical to those used in Experiment 1.

#### 3.1.3. Design

The design was the same as in Experiment 1.

#### 3.1.4. Procedures

Experiment 2 followed the same procedures as Experiment 1, with the study phase materials presented twice, allowing for a second round after the initial completion. A total of 100 “category–exemplar” word pairs were studied.

### 3.2. Results

#### 3.2.1. Recall Performance of Speakers

**Figure 3:**
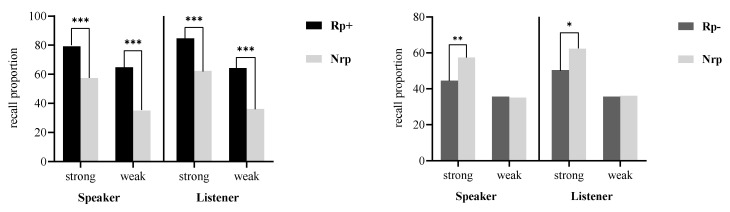
The left subfigure shows the practice effects (Rp+ vs. Nrp); the right subfigure shows the RIF (Rp− vs. Nrp). **Note:** *** *p* < 0.001; ** *p* < 0.01; * *p* < 0.05.

Table 2 shows the recall performance of speakers and listeners in Experiment 2. Figure 3 illustrates the results of the *t*-tests in Experiment 2.

For practice effects, a 2 (item strength: strong, weak) × 2 (item type: Rp+, Nrp) repeated-measures ANOVA showed significant main effects for item type (*F* (1, 27) = 32.87, *p* < 0.001, *η_p_^2^* = 0.55) and item strength (*F* (1, 27) = 43.59, *p* < 0.001, *η_p_^2^* = 0.62), with a non-significant interaction (*F* (1, 27) = 2.82, *p* = 0.104, *η_p_^2^* = 0.09). Planned paired *t*-tests confirmed higher recall for Rp+ over Nrp items at both strengths, indicating practice effects (strong: *t* (27) = 3.87, *p* < 0.001, Cohen’s *d* = 0.73; weak: *t* (27) = 6.60, *p* < 0.001, Cohen’s *d* = 1.25).

For RIF, a 2 (item strength: strong, weak) × 2 (item type: Rp−, Nrp) repeated-measures ANOVA revealed a significant main effect for item strength (*F* (1, 27) = 26.50, *p* < 0.001, *η_p_^2^* = 0.50) and a marginally significant effect for item type (*F* (1, 27) = 3.98, *p* = 0.056, *η_p_^2^* = 0.06), with a significant interaction (*F* (1, 27) = 8.05, *p* = 0.0085, *η_p_^2^* = 0.23.). Planned paired *t*-tests showed RIF with strong items (*t* (27) = 3.09, *p* = 0.005, Cohen’s *d* = 0.58, B_10_ = 17.72), but no RIF with weak items (*t* (27) = −0.17, *p* = 0.868, Cohen’s *d* = −0.03, B_10_ = 0.18), indicating RIF is present only with strong items.

#### 3.2.2. Recall Performance of Listeners

For practice effects, in a 2 (item strength: strong, weak) × 2 (item type: Rp+, Nrp) repeated-measures ANOVA, significant main effects were found for item type (*F* (1, 27) = 81.55, *p* < 0.001, *η_p_^2^* = 0.75) and item strength (*F* (1, 27) = 71.90, *p* < 0.001, *η_p_^2^* = 0.73), with no significant interaction (*F* (1, 27) = 1.95, *p* = 0.174, *η_p_^2^* = 0.07). Planned paired *t*-tests showed higher recall for Rp+ over Nrp items at both strengths (strong: *t* (27) = 6.84, *p* < 0.001, Cohen’s *d* = 1.29; weak: *t* (27) = 7.57, *p* < 0.001, Cohen’s *d* = 1.43), indicating practice effects.

For SS-RIF, another 2 (item strength: strong, weak) × 2 (item type: Rp−, Nrp) repeated-measures ANOVA revealed significant main effects for item type (*F* (1, 27) = 4.78, *p* = 0.038, *η_p_^2^* = 0.15) and item strength (*F* (1, 27) = 77.49, *p* < 0.001, *η_p_^2^* = 0.74), and a significant interaction (*F* (1, 27) = 4.38, *p* = 0.046, *η_p_^2^* = 0.14). Planned paired *t*-tests indicated SS-RIF in strong items (*t* (27) = 2.49, *p* = 0.019, Cohen’s *d* = 0.47, B_10_ = 5.26), but not in weak items (*t* (27) = 0.13, *p* = 0.897, Cohen’s *d* = 0.02, B_10_ = 0.22), demonstrating SS-RIF only with strong items.

#### 3.2.3. The Effect of Restudy on RIF and SS-RIF

In Experiments 1 and 2, RIF and SS-RIF were observed only for strong items. To examine if restudy influenced RIF and SS-RIF for strong items, we conducted independent sample *t*-tests to compare the magnitude of RIF and SS-RIF between the two experiments. This analysis aimed to determine if there was a difference in RIF and SS-RIF magnitudes based on whether the material was studied once or twice. The magnitude of RIF and SS-RIF was quantified by calculating the difference in recall performance between Nrp and Rp− items for speakers and listeners, respectively. The results indicated no significant change in RIF for speakers or SS-RIF for listeners across experiments (RIF: *t* (54) = −0.23, *p* = 0.816, Cohen’s *d* = −0.06; SS-RIF: *t* (54) _(SS-RIF)_ = 0.09, *p* = 0.928, Cohen’s *d* = 0.02). This suggested that restudy did not significantly affect RIF or SS-RIF performance.

### 3.3. Discussion

Experiment 2 replicated Experiment 1’s findings of RIF and SS-RIF occurring only with strong items, despite enhanced memory performance. Similar patterns of RIF and SS-RIF across item strength imply a shared mechanism, likely inhibition mechanism in nature. Comparative analysis showed no significant differences in the magnitude of RIF or SS-RIF between the experiments, warranting further investigation into the inhibition mechanism’s role.

## 4. General Discussion

In this study, two experiments investigated the effect of item strength on SS-RIF to explore its mechanisms. Experiment 1, using the SS-RIF paradigm, revealed RIF and SS-RIF in strong items only. Experiment 2, with increased study sessions, replicated these findings, aligning with “interference dependence” and supporting the inhibition mechanism for both RIF and SS-RIF. The consistent performance across experiments suggests similar underlying mechanisms for RIF and SS-RIF.

Firstly, this study bolstered the inhibition theory’s account of SS-RIF and RIF, with RIF evident solely among strong items, corroborating prior research [1,13,25]. Listeners mirrored this pattern, showing SS- RIF exclusively for strong items. Contrary to the blocking theory’s anticipation of RIF in both item strengths due to strengthened Rp+ items, our findings revealed enhanced recall for Rp+ items without diminished performance for Rp− items in weak items. This outcome challenges the general applicability of the blocking mechanism to RIF and SS-RIF.

Such results, where only strong items exhibited RIF and weak items did not exhibit RIF, were consistent with the study by Anderson et al. [1]. Their findings on item strength are critical evidence in support of “interference dependence.” Anderson originally proposed the inhibition theory of RIF, which essentially aimed to resolve the competition from non-target items during the retrieval phase. For strong items, it is more likely that they will be activated, leading to competition during the retrieval phase, where the inhibition mechanism needs to operate effectively. Weak items are inherently difficult to activate sufficiently to compete, so there is no need for the inhibition mechanism to work. For SS-RIF, researchers have suggested that SS-RIF and RIF have similar mechanisms [18,19]. The reason that listeners forget the same content as speakers is that the listener “covertly retrieves” the content of the speaker’s words, whether through “covert retrieve” leading to inhibition of Rp− items, blocking by strengthening Rp+ items, or a combination. Our study clarifies this by demonstrating SS-RIF in strong items only, suggesting that SS-RIF is likely an effect of the inhibition mechanism.

Secondly, in our examination of SS-RIF and RIF, we build upon the comparative analysis conducted by Abel and Bäuml [20]. They posited that SS-RIF is more susceptible to the blocking effect and less influenced by the inhibition process than RIF, a conclusion grounded in the two-factor theory framework. However, our study’s findings challenge this conclusion, suggesting that the inhibition and blocking mechanisms may not always operate simultaneously. Contrary to the expectation that both mechanisms would be active concurrently, in our results, neither RIF nor SS-RIF was observed for weak items across both experiments. This observation prompts a reconsideration of the two-factor theory’s applicability in all contexts. We do not aim to refute the theory, but rather to highlight potential limitations in its generalizability, particularly in light of our findings. The operation of RIF mechanisms could differ between speakers and listeners, influenced by contextual factors. For instance, under conditions favoring inhibition, similar patterns may emerge in both. However, when inhibition and blocking operate together, listeners’ inhibition might be reduced. Additionally, we suggest that experimental conditions could selectively enhance the blocking mechanism, indicating a need for further research.

Thirdly, certain findings challenged the property of “interference dependence”. Williams and Zacks [15] challenged “interference dependence” by replicating Anderson et al. [1] and finding RIF in both strong and weak items, suggesting a primary role for blocking over inhibition. However, in their Experiment 2, strong items showed greater enhancement of Rp+, while weak items exhibited greater impairment for Rp−, contradicting the blocking theory’s expectations. The inconsistencies indicate that blocking theory may not solely explain the observed RIF in Williams and Zacks’s study, implying a potential dual contribution from both inhibition and blocking mechanisms. Extended presentation times, as in Williams and Zacks’s study (8 s per exemplar versus Anderson’s 5 s) and longer retrieval times (10 s), likely strengthen item–cue associations, facilitating blocking of non-target items and contributing to RIF in weak items. Raaijmakers and Jakab [26] also found RIF with low-frequency items through non-competitive retrieval, suggesting that weak items can induce RIF via blocking given certain conditions. This points to the possibility of both blocking and inhibition mechanisms operating simultaneously, especially with stronger item–cue associations.

Jakab and Raaijmakers [16] similarly did not find that strong items exhibit a larger RIF effect than weak items. While memory performance differed for items at different positions within a category, there was no difference in RIF across these positions. Additionally, restudying improved memory performance, but the magnitude of RIF was equivalent between the single-study and double-study conditions. These results did not support interference dependence, but were consistent with the results of the present study. None of them reflected the effect of memory performance on SS-RIF and RIF. Our analysis suggests the result is related to the inhibitory mechanism’s operation, as described by Saunders and MacLeod [27], where inhibitory control restricts activation spread at the category level. The competitive level correlates with non-target item activation, and competition strength determines the degree of non-target inhibition.

Anderson et al. [1] categorized items by taxonomic frequency, with higher-frequency items more likely to activate and compete with targets, exhibiting RIF due to greater susceptibility to inhibition. In Jakab and Raaijmakers’s study [16], all items were of moderate taxonomic frequency. Based on category cues, the extent to which these items can be activated should be equivalent. Although the memory performance of items in different category positions varied, the Rp+ and Rp− items were similar across all category positions. This means that there was no difference in the level of activation that they could generate, and the competitive strength of non-target items remained unchanged. Therefore, it became difficult to demonstrate variations in the magnitude of RIF at different positions.

Consistent with the study by Jakab and Raaijmakers [16], the present study also failed to detect an impact of restudy on RIF and SS-RIF. Jakab and Raaijmakers [16] proposed that restudy, by enhancing memory performance, should lead to increased item strength and consequently more pronounced RIF following restudy. However, their findings did not meet these expectations. Similarly, a comparison between Experiment 2 and Experiment 1 in this study did not reveal any differences in the magnitude of RIF or SS-RIF between the two experiments. This is a noteworthy issue, because the RIF and SS-RIF performance of both strong and weak items in both experiments is in line with “interference dependence,” yet the results before and after restudy do not conform to “interference dependence.” We propose two hypotheses to explain these findings. One is that the “item strength” associated with restudy is distinct from the “item strength” related to exemplar taxonomic frequency. Extensive research has shown that the “item strength” tied to taxonomic frequency is closely linked to the competitive strength among items [23]. The “item strength” enhanced by restudy may not be related to the competitive strength of items within the RIF paradigm; thus, despite improved memory performance following restudy, no change in RIF was observed. Another hypothesis is that the intensity of restudy is insufficient. Although memory performance for items improved in both this study and that of Jakab and Raaijmakers [16] through restudy, this enhancement did not reach a qualitative threshold that could trigger a substantial increase in competitive strength among items, and accordingly no change in RIF was observed. However, if the intensity of restudy is sufficient, an increase in RIF could still be observed. Future research can adjust the conditions of restudy to test these hypotheses.

Limitations. This study provides support for the inhibition mechanism of SS-RIF and RIF from the perspective of item strength. However, our findings do not negate the potential role of the blocking mechanism. Drawing on comparisons with prior research, we suggest that there may be specific boundary conditions under which the blocking mechanism operates, a topic ripe for exploration in future studies. The use of cued recall in our study, while comprehensive for SS-RIF examination, presents challenges in controlling for output interference. Nonetheless, evidence suggests that recall order does not influence RIF [12,28], implying that our conclusions are robust to this limitation. Recruitment via advertisements resulted in a predominantly female participant pool and limited the sample to university students, which are limitations of this study. Future studies should broaden the sample of participants to better understand the mechanisms of SS-RIF and RIF across different groups.

## 5. Conclusions

In conclusion, the present study’s findings on RIF for both speakers and listeners support interference dependence, offering compelling evidence for the inhibition mechanism in both SS-RIF and RIF.

## Figures and Tables

**Table 1:** Means ± SDs for recall performance in Experiment 1.

	Item Strength	Rp+	Rp−	Nrp
Speaker	Strong	86.31 ± 16.39	35.12 ± 25.80	46.43 ± 19.30
	Weak	61.31 ± 22.70	25.00 ± 19.51	29.17 ± 15.13
Listener	Strong	82.14 ± 13.55	35.12 ± 25.39	47.62 ± 18.54
	Weak	63.10 ± 15.78	27.38 ± 18.27	29.76 ± 15.78

**Table 2:** Means ± SDs for recall performance in Experiment 2.

	Item Strength	Rp+	Rp−	Nrp
Speaker	Strong	79.17 ± 19.58	44.64 ± 31.77	57.44 ± 24.36
	Weak	64.88 ± 19.55	35.71 ± 22.55	35.12 ± 21.56
Listener	Strong	84.67 ± 15.76	50.45 ± 24.65	62.35 ± 21.05
	Weak	64.29 ± 24.80	35.71 ± 16.01	36.09 ± 14.56

## Data Availability

The data are available from the corresponding author on reasonable request.
